# Postcranial heterochrony, modularity, integration and disparity in the prenatal ossification in bats (Chiroptera)

**DOI:** 10.1186/s12862-019-1396-1

**Published:** 2019-03-12

**Authors:** Camilo López-Aguirre, Suzanne J. Hand, Daisuke Koyabu, Nguyen Truong Son, Laura A. B. Wilson

**Affiliations:** 1 PANGEA Research Centre, School of Biological, Earth & Environmental Sciences, University of New South Wales, Sydney, NSW 2052 Australia; 2 University Museum, University of Tokyo, Tokyo, Japan; 3 Department of Humanities and Sciences, Musashino Art University, Tokyo, Japan; 4 Department of Vertebrate Zoology, Institute of Ecology and Biological Resources, Vietnam Academy of Sciences and Technology, Hanoi, Vietnam; 5 Vietnam Academy of Science and Technology, Graduate University of Science and Technology, Hanoi, Vietnam

**Keywords:** Sequence heterochrony, Postcranial development, Prenatal growth, Modularity, Integration, Ontogeny

## Abstract

**Background:**

Self-powered flight is one of the most energy-intensive types of locomotion found in vertebrates. It is also associated with a range of extreme morpho-physiological adaptations that evolved independently in three different vertebrate groups. Considering that development acts as a bridge between the genotype and phenotype on which selection acts, studying the ossification of the postcranium can potentially illuminate our understanding of bat flight evolution. However, the ontogenetic basis of vertebrate flight remains largely understudied. Advances in quantitative analysis of sequence heterochrony and morphogenetic growth have created novel approaches to study the developmental basis of diversification and the evolvability of skeletal morphogenesis.

Assessing the presence of ontogenetic disparity, integration and modularity from an evolutionary approach allows assessing whether flight may have resulted in evolutionary differences in the magnitude and mode of development in bats.

**Results:**

We quantitatively compared the prenatal ossification of the postcranium (24 bones) between bats (14 species), non-volant mammals (11 species) and birds (14 species), combining for the first time prenatal sequence heterochrony and developmental growth data. Sequence heterochrony was found across groups, showing that bat postcranial development shares patterns found in other flying vertebrates but also those in non-volant mammals. In bats, modularity was found as an axial-appendicular partition, resembling a mammalian pattern of developmental modularity and suggesting flight did not repattern prenatal postcranial covariance in bats.

**Conclusions:**

Combining prenatal data from 14 bat species, this study represents the most comprehensive quantitative analysis of chiropteran ossification to date. Heterochrony between the wing and leg in bats could reflect functional needs of the newborn, rather than ecological aspects of the adult. Bats share similarities with birds in the development of structures involved in flight (i.e. handwing and sternum), suggesting that flight altriciality and early ossification of pedal phalanges and sternum are common across flying vertebrates. These results indicate that the developmental modularity found in bats facilitates intramodular phenotypic diversification of the skeleton. Integration and disparity increased across developmental time in bats. We also found a delay in the ossification of highly adaptable and evolvable regions (e.g. handwing and sternum) that are directly associated with flight performance.

**Electronic supplementary material:**

The online version of this article (10.1186/s12862-019-1396-1) contains supplementary material, which is available to authorized users.

## Background

Pterosaurs, birds and bats are the only vertebrates capable of self-powered flight (herein refered to as flight, [1]). However, the phylogenetic relationships between these groups and their position in the evolutionary history of vertebrates have shown that this feature evolved in each group independently, as a result of convergent evolution [1, 2]. Historically, the convergent evolution of vertebrate flight was asynchronous, with pterosaurs evolving flight first (≈240 Mya), followed by birds (≈140 Mya), and bats (≈60 Mya) [3–5]. Flight was a key innovation that provided a major ecological opportunity for these groups, allowing them to diversify into a vast range of previously empty niches [6]. As a result, both birds and bats are some of the most speciose groups of living vertebrates. It is estimated that pterosaurs were also abundant and diverse before their extinction, with over 160 fossil species described [7].

It has been suggested that all three groups share some morphological and physiological adaptations in body regions that are vital for the kinematics of flight [8]. Reduction of cortical bone thickness [9], increased bone density [8], morphological changes in the pectoral girdle [10], and elongation of forelimb bones are some of the adaptations that these groups share [11]. It has also been found that all three groups have relatively small genomes when compared to their respective close relatives [3], suggesting that constricted genome sizes has been evolutionarily correlated with the evolution of self-powered flight in vertebrates [3, 12].

The relatively abundant fossil record of pterosaurs and birds has facilitated the study of the evolution of flight in these groups. Bat flight evolution, on the other hand, has been comparably more difficult to study due to the incompleteness of the fossil record, limiting the scope for research [13–22]. Studying how the development of organisms reflects their evolutionary history [23–28], represents an alternative to assess the extent of interspecific variation in postcranial morphology over the course of ontogeny, data that could provide a baseline for further study into the evolution of mammalian flight [22, 29].

Based on previous studies that used ossification to study morphogenesis, we now know that the development of specific traits is not homogenous across taxa that share those features [30–32]. This has been demonstrated for both analogous and homologous traits [33]. Comparisons of ossification sequences have shown that the interaction between ontogeny and ecology has a domino effect on the modification and specialisation of the mammalian skeleton [34, 35]. Nevertheless, previous studies have suggested that it may not always be possible to differentiate between functional and phylogenetic developmental fluctuations [36]. The delimitation between ontogenetic changes reflecting a phylogenetic signal and changes reflecting ecomorphological adaptations is not clear when analysing developmental sequences without testing a specific hypothesis [36].

There are two general approaches to study changes in morphology during development: sequence heterochrony and developmental growth analyses [37]. Sequence heterochrony analyses document variations in the timing and order in which a group of traits starts developing, permitting the study of developmental events that are not explicitly characterized by size or shape [37]. Developmental growth refers to differences in the timing of growth onset, and growth rate that different traits experience during the development of an individual [37]. Studying sequence heterochrony has been particularly useful to elucidate developmental differences in patterns of ossification in a wide range of vertebrate taxa [35, 37–41]. Among vertebrates, mammals exhibit the widest range of skeletal specialisations for different locomotor and feeding strategies [23–25, 42]. As a result, mammals have successfully adapted to more ecosystems than any other vertebrate group [6, 14, 43]. Sequence heterochrony studies of skeletal ossification in mammals have suggested an ontogenetic basis for the functional adaptability of the postcranium [23, 24, 44, 45]. Sequence heterochrony in mammals has also been linked to both phylogeny and life history [36, 44, 45].

Developmental growth studies have helped to elucidate evolutionary changes in ontogenetic trajectories across lineages [23, 24, 33, 35, 46]. By focusing on the morphological changes across the development of a structure, it is possible to recreate the relationship between shape and size and how it responds to ecological, genetic and ontogenetic constrains [47–50]. This principle has been applied to the evolutionary history of different taxa, reconstructing ancestral states and historical divergences and diversifications, providing a developmental perspective to the study of evolution [51–54].

Prenatal and postnatal development have been found to be drastically dissimilar processes for some species [55–58], experiencing different selective pressures [56, 58], and resulting in differential effects on morphological disparity at adulthood [34, 48, 56, 58]. A prenatal developmental basis has been suggested for the phenotypic diversity in some mammal species, proposing that prenatal development shows more variability as it does not experience strong environmental changes [54, 55]. Moreover, a link between altriciality and the capacity of a system to evolve (i.e. evolvability; [26]) has been proposed, in which incomplete development at birth and extended developmental times have been hypothesised to facilitate adaptability [56, 58].

Another aspect of mammalian evolution that has been increasingly studied is how the interaction between morphological disparity and integration shape the phenotypic evolution of a clade [47, 59–61]. Morphological disparity refers to the phenotypic variability within a set of individuals, whereas morphological integration indicates a correlation in the morphological variation of a set of traits [62, 63]. Recent quantitative studies have examined the role that the interaction between disparity and integration could play in the evolutionary history of a group, leading to two, mutually-exclusive hypotheses tractable to testing: 1) high levels of integration restrains disparity, canalising all phenotypes to a similar state [59], or 2) high levels of integration create an “evolutionary buffer” that facilitates disparity, leading to phenotypic diversification [59]. These two hypotheses help to define the range of possible forms that the disparity-integration interaction can take in biological systems. Furthermore, integration can create groups of traits that show high within-group correlations, but that are weakly connected and therefore relatively independent from other groups (i.e. modularity; [63–65]). Consequently, one could expect that modularity would vary in its configuration as integration of the entire system is higher, as all traits are strongly correlated.

Despite increased interest in the morphological development and ontogeny of mammals [22, 23, 38, 47, 66, 67], many questions regarding the ontogenetic basis of modern phenotypic diversity remain unanswered. Bats are an excellent example of ecomorphological diversity within Mammalia, having a wide range of dietary specialisations [68], and exhibiting a range of locomotor strategies [69]. Bats show a unique, highly derived postcranial body plan adapted to flight (e.g. elongated forelimbs, reduced bone cortical thickness, specialised pectoral girdle morphology) [8–11], but different bat species are also capable of walking and in some extreme cases swimming [70–72]. To date, few studies have focused on quantifying and interpreting heterochrony of the postcranium in bats [73, 74]. Koyabu and Son [36] showed that bats have accelerated ossification of the phalanges of the hindlimbs and thumb during prenatal development, and suggested that this pattern could be a functional response to the need to continuously attach to their mothers and to the substrate of their roosts. This is particularly relevant in bats because newborns are incapable of flying for several weeks or up to several months after birth [36]. Given that vertebrate flight is polyphyletic, it is possible that the processes underlying postcranial development in bats converged with those of other flying vertebrates. Consequently, bat postcranial ossification could diverge from a more phylogenetically constrained mammalian development to a more functionally convergent flying vertebrate development. However, our understanding of the developmental basis of vertebrate flight remains limited.

Combining sequence heterochrony and metric growth data, this study compared the prenatal ossification of the postcranium in bats, birds and non-volant mammals, evaluating whether flight may have shaped the magnitude and mode of postcranial development in bats compared to non-volant mammals and birds. We used the most comprehensive sampling of prenatal bats to date. We quantify the levels of disparity and integration across bones during ossification, to assess whether integration restrains or promotes disparity during the development of bats. Considering that flight and the associated morphological specialisations are exclusive to bats among mammals, we expect to find a shift in the postcranial development of bats compared to non-volant mammals, showing some similarities with other flying vertebrates. Also, based on the embryological origin and functional differences of different regions of the postcranium, we expect to find evidence of ontogenetic modularity. As a result, we expect a positive correspondence between magnitudes of disparity and integration within each module, as well as differences in levels of disparity and integration between modules.

## Methods

### Data collection

**Fig. 1:**
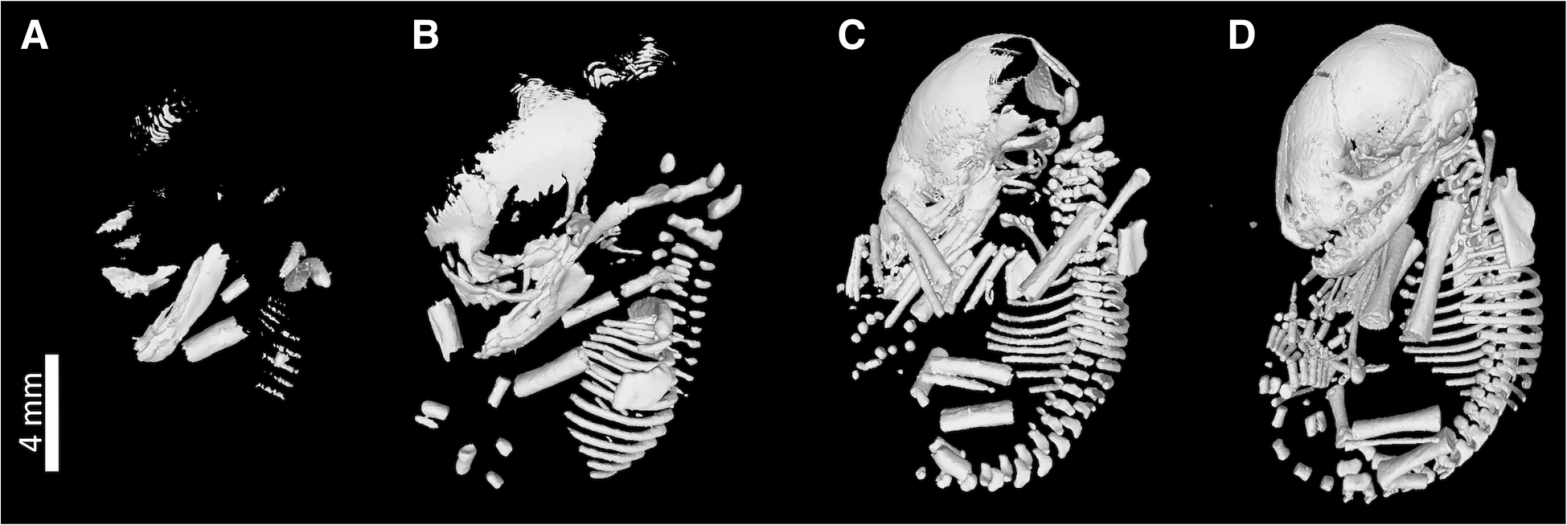
3D virtual models of ontogenetic series of *H. blanfordi*, representing the samples from which raw measurements were taken from postcranial elements. Specimens represent different developmental stages: Stage 1 (**a**), stage 3 (**b**), stage 8 (**c**), and stage 10 (**d**). Stage 1 is characterised by ossification of the humerus, clavicle and ribs; stage 3 by ossification of ilium and tibia; stage 8 by ossification of manual phalanges, and stage 10 by ossification of carpals

To study prenatal postcranial ossification in bats, 66 specimens representing developmental series of 11 bat species (*Aselliscus dongbacana*, *A. stoliczkanus*, *Cynopterus sphinx*, *Hesperoptenus blanfordi*, *Hipposideros larvatus*, *Kerivoula hardwickii, Miniopterus schreibersii*, *Myotis* sp*.*, *Rhinolophus pearsoni, R. pusillus*, *R. thomasi*) were sampled, comprising five families and members from both chiropteran suborders Yinpterochiroptera and Yangochiroptera (Table 1). Specimens were collected and prepared in Vietnam for a study of sequence heterochrony in bats [36], and stored in 70% ethanol. Grey scale images of specimens were acquired using a microfocal X-ray CT system at the University Museum, University of Tokyo (TXS225-ACTIS, TESCO, Tokyo) with 70 kV source voltage and μ114 A source currents at a resolution of 36 μm (Fig. 1). Segmentation of the skeleton from other tissues was performed using the thresholding tool in MIMICS v. 20 software (Materialise NV), using the bone (CT) predefined set as a basis. Finer thresholding to separate CT-values of osseous and non-osseous structures followed the Half Maximum Height method (HMH) [75]. This technique did not allow us to reconstruct cartilaginous tissue during the growth of the skeleton, limiting our capacity to describe the growth of non-osseous tissue in the bone.

**Table 1:** List of mammal and bird species analysed in this study

Class	Order	Family	Species	Source
Mammalia	Chiroptera	Hipposideridae	*Aselliscus dongbacana*	This study (*n* = 14)
			*Aselliscus stoliczkanus*	This study (*n* = 16)
			*Hipposideros larvatus*	This study (*n* = 4)
		Pteropodidae	*Cynopterus sphinx*	This study (n = 4)
			*Rousettus amplexicaudatus*	[23]
		Rhinolophidae	*Rhinolophus pearsoni*	This study (n = 1)
			*Rhinolophus pusillus*	This study (n = 1)
			*Rhinolophus thomasi*	This study (*n* = 10)
		Vespertilionidae	*Hesperoptenus blanfordi*	This study (*n* = 5)
			*Kerivoula hardwickii*	This study (*n* = 7)
			*Myotis* sp.	This study (*n* = 2)
			*Myotis ater*	[36]
			*Myotis lucifugus*	[36]
		Miniopteridae	*Miniopterus schreibersii*	This study (n = 2)
	Rodentia	Muridae	*Rattus norvegicus*	[36]
			*Meriones unguiculatus*	[36]
			*Mus musculus*	[36]
			*Rhabdomys pumilio*	[36]
		Cricetidae	*Peromyscus melanophrys*	[36]
			*Mesocricetus auratus*	[36]
		Octodontidae	*Octodon degus*	[36]
	Eulipotyphla	Soricidae	*Cryptotis parva*	[36]
		Talpidae	*Talpa europaea*	[36]
	Cetartiodactyla	Bovidae	*Bos taurus*	[36]
		Suidae	*Sus scrofa*	[36]
Aves	Galliformes	Phasianidae	*Meleagris gallopavo*	[77]
			*Coturnix coturnix*	[77]
			*Gallus gallus*	[77]
	Struthioniformes	Struthionidae	*Struthio* sp.	[77]
	Casuariiformes	Casuariidae	*Dromaius novaehollandiae*	[77]
	Anseriformes	Anatidae	*Somateria mollissima*	[77]
			*Anas platyrhynchos*	[77]
			*Cairina moschata*	[77]
	Pelecaniformes	Phalacrocoracidae	*Phalacrocorax auritus*	[77]
	Charadriiformes	Laridae	*Sterna hirundo*	[77]
			*Larus ridibundus*	[77]
			*Larus argentatus*	[77]
			*Larus canus*	[77]
		Stercorariidae	*Stercorarius skua*	[77]

Ossification sequence data of 24 postcranial elements were recorded by analysing the 3D virtual models of the postcranial skeleton using MIMICS v. 20 software (Materialise NV). Raw models of scanned specimens were thresholded to generate the virtual models of the postcranial skeleton (Fig. 1). Ossification sequences for three additional species were compiled from previous studies (Additional file 1: Figure S1): *Rousettus amplexicaudatus* [23], *Myotis ater* [36] and *M. lucifugus* [76]. Prenatal ossification sequences of 11 non-volant mammal species and 14 bird species were consolidated based on previously published studies [36, 77]. Our sample included three orders, eight families of non-volant mammals, and six orders and seven families of birds.

### Sequence heterochrony

For each specimen, the ossification level of each bone was codified as one of three categories: unossified, ossification onset, or partly ossified. To consolidate the ossification sequence of each species, we followed a modified absolute rank *r* standardisation implemented in previous studies [23, 24, 34, 78, 79]. The traditional approach standardises the absolute rank *r* by the maximum number of ranks, which has been shown to result in high variability in the relative rank of the first bones to ossify [36]. To address this, the relative rank of each species was scaled from 0 (i.e. earliest ossification event) to 1 (i.e. latest ossification event), removing possible interspecific differences in the maximum rank number (see [74]). Only species with well-resolved developmental series (i.e. ≥ 4 ranks) were included in further analyses.

To compare the prenatal ossification sequences of bats with both non-volant mammals and flying vertebrates, we controlled for anatomical differences between birds and mammals, standardising a unique anatomical nomenclature of homologous structures. Consequently, the avian furcula was matched with the mammalian clavicle. Given the complex structure of the avian synsacrum, the ossification of the avian sacral vertebrae was paired with the lumbar and sacral mammalian vertebrae. Finally, in order to visualise developmental differences across groups, we performed a Principal Component Analysis (PCA) of the ossification sequences of all 39 species pooled as bats, birds and non-volant mammals. All analyses were performed with PAST 3.18 [80].

### Metric growth

3D virtual reconstructions of the postcranial skeleton of all 66 specimens were used to collect a total of 25 linear measurements (Table 2). Measurements were obtained from 3D renderings of the virtual models using the *measure distance* tool in MIMICS v. 20 software (Materialise NV). Measurements were logarithmically transformed prior to analysis. Missing values were replaced using linear interpolation with the *na.approx* function of the R package *zoo* version 1.8 [81]. Mean values of each measurement were used to create a single developmental growth sequence for each species.

**Table 2:** Description of 25 linear measurements of the postcranial skeleton of bat fetuses used in this study

Measurement	Acronym	Description	Module
Crown-Rump Length	CRL	Length from the top of the head to bottom of torso	–
Humeral Length	HL	Left humerus length	3, 4, 6, 8
Clavicular length	CL	Left clavicle length	2, 6, 8
Scapular length	SL	Left scapula length	2, 6, 8
Femoral length	FL	Left femur length	3, 7, 8
Rib length	RiL	First left rib length	2, 9
Tibial length	TL	Left tibia length	3, 7, 8
Fibular length	FiL	Left fibula length	3, 7, 8
Radial length	RL	Left radius length	3, 4, 6, 8
Ulnar length	UL	Left ulna length	3, 4, 6, 8
Sternum length	StL	Sternum length	2, 8
Manual phalange length	MPL	Proximal phalange of second digit of left forelimb	3, 5, 6, 8
Pedal phalange length	PPL	Proximal phalange of first digit of left hindlimb	3, 7, 8
Metacarpal length	McL	Metacarpus of second digit of left forelimb	3, 5, 6, 8
Metatarsal length	MtL	Metatarsal of first digit of left hindlimb	3, 7, 8
Tarsal length	TaL	Length of calcaneus of left hindlimb	3, 7, 8
Carpal length	CaL	Length of scaphoid of left forelimb	3, 5, 6, 8
Cervical vertebral width	CvL	C1 vertebral width	1, 2, 9
Thoracic vertebral width	TvL	T1 vertebral width	1, 2, 9
Lumbar vertebral width	LvL	L1 vertebral width	1, 2, 9
Sacral vertebral width	SvL	S1 vertebral width	1, 2, 9
Caudal vertebral width	CavL	vertebral width of first caudal vertebra	1, 2, 9
Ilium length	IlL	Length from top of iliac crest to the edge of triradiate joint	2, 7, 8
Ischium length	IsL	Length from bottom of ramus to edge of triradiate joint	2, 7, 8
Pubis bone length	PuL	Length from bottom of ramus to edge of triradiate joint	2, 7, 8

Numbers indicate the modularity hypotheses where each bone was included (see Table 3 for number coding)

For bone categories representing more than a single bone (e.g. ribs and vertebrae) we always measured the same bone, usually being the one first to ossify within the category (see Table 2 for specifications). To control possible bilateral asymmetry due to developmental instability in our data, we measured bones only on the left side for all specimens. Since all analyses were performed at ordinal level, all species were included in further analysis, including *R. pearsoni* and *R. pusillus* that were represented only by one individual each.

### Developmental modularity

To assess the presence of correlation between prenatal ossification ranks we conducted a neighbour-joining clustering analysis based on the scaled ossification ranks of each bone, bootstrapping each node with 10,000 permutations [36]. Kendall’s τ was used to test modularity in prenatal ossification ranks for bats, birds and non-volant mammals separately, performing pairwise comparisons between all 24 bones based on their relative ossification ranks. Following previous studies [59], seven different functional and anatomical modularity hypotheses were tested: 1) vertebral column, 2) axial skeleton, 3) appendicular skeleton, 4) armwing, 5) handwing, 6) forelimb and 7) hindlimb. Additionally, to explore the ontogenetic basis of modularity, we developed two new hypotheses based on the embryological origin of the bone: 8) abaxial skeleton or lateral plate mesoderm (LPM)-derived, and 9) primaxial skeleton or somite-derived (see Table 2 for specifications, [82–84]). All hypotheses were evaluated for Chiroptera, non-volant mammals and birds.

Finally, to visualise the similarities between bone growth patterns informed by the modules obtained in the two previous analyses, we performed a PCA grouping all 24 bones into the best supported modularity model. All analyses were performed with PAST 3.18 [80].

### Developmental disparity and integration

Morphological integration was interpreted as the relative eigenvalue standard deviation (i.e. eigenvalue dispersion) of a PCA of all 24 bones for all 66 bat specimens. With this approach, high levels of dispersion mean that few eigenvectors account for a high proportion of variance, indicating strong integration [85]. Morphological disparity was measured as the statistical variance of the linear measurements of each bone. To test differences in disparity and integration between hypothetical developmental modules we averaged the values obtained for all bones within each module. Finally, to describe temporal shifts in disparity and integration across the prenatal development of Chiroptera, we staged all bat specimens based on criteria previously published for different bat species [86–90]. Since CS staging systems have not been developed for nine of the 11 species in the prenatal sample, specimens were classified into one of 10 developmental stages (1–10), based on the development of external features, bone ossification sequence and crown to rump length (CRL, Fig. 1). Linear Discriminant Function Analysis (LDA) was performed to statistically assess the accuracy of our staging system and visualise the spatial separation between the stages (Additional file 2 Figure S2). By combining both datasets we circumvent the issue of establishing discrete categories along a continuous morphometric dimension, informing our staging system with a discrete component of development. Generalised Linear Model (GLM) was used to test the correlation between integration and disparity across bones and developmental time and PCA to visualise the distance between species in the developmental space in PAST 3.18 [80].

The effect of uneven bat species representation across developmental stages on values of disparity and integration was assessed with Pearson’s correlations. Also, Pearson’s correlations were used to test whether the number of specimens per developmental stage influences values of disparity and integration. Uncertainty in the values of disparity and integration was assessed by estimating 95% confidence intervals with 10,000 bootstrap replicates using the boot package version 1.3 in R [91].

## Results

### Sequence heterochrony

**Fig. 2:**
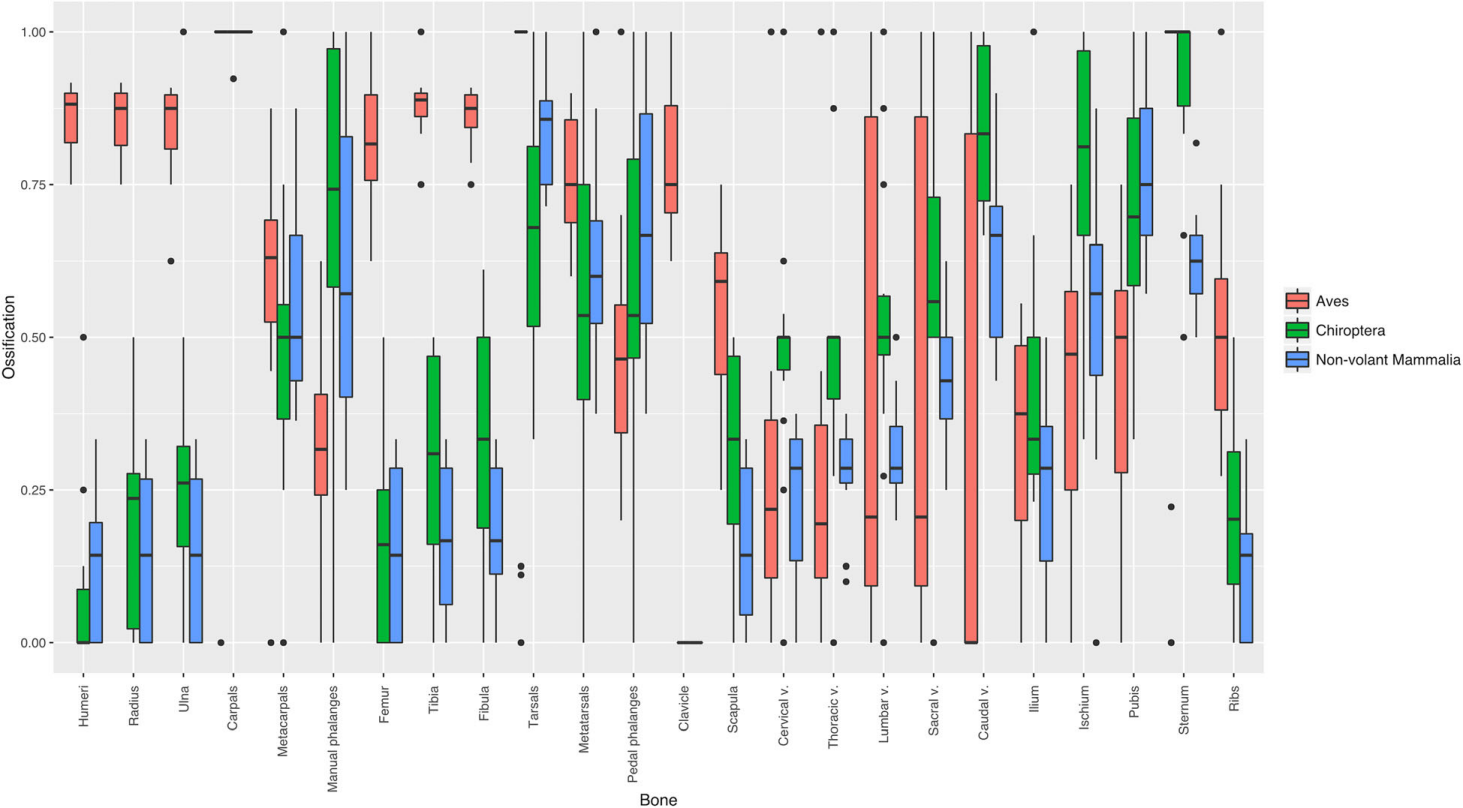
Relative timing of onset of ossification (ranks) of 24 postcranial bones in Aves (red), Chiroptera (green), and non-volant mammals (blue). Standardised ranking of bone ossification onset ranges from 0 (first to start ossification) to 1 (last to start ossification)

General patterns of sequence heterochrony were evident across the chiropteran skeleton. Bones of the stylopod (proximal section) and zeugopod of both limbs, and the pectoral girdle are the first ones to ossify, followed by bones of the autopod (distal section) of both limbs and all but the caudal vertebrae of the spinal column (Fig. 2). Carpals, caudal vertebrae, ischium and sternum are the last bones to ossify. Metatarsals and pedal phalanges showed the highest variance in their relative ranking, whereas the clavicle was invariably the first bone to ossify. Compared to the other groups, postcranial ossification sequence in bats have similarities with both non-volant mammals and birds (Fig. 2). Vertebral column ossification in bats is delayed compared to non-volant mammals and birds, showing a distinctive developmental process in this region unique to bats, a pattern also found for the ischium. In contrast, bats and non-volant mammals show an early ossification of the stylopod and zeugopod of both limbs, compared to birds. Bats and birds shared a delay in the ossification of the sternum, compared to non-volant mammals. All groups had a late ossification of the carpals.

**Fig. 3:**
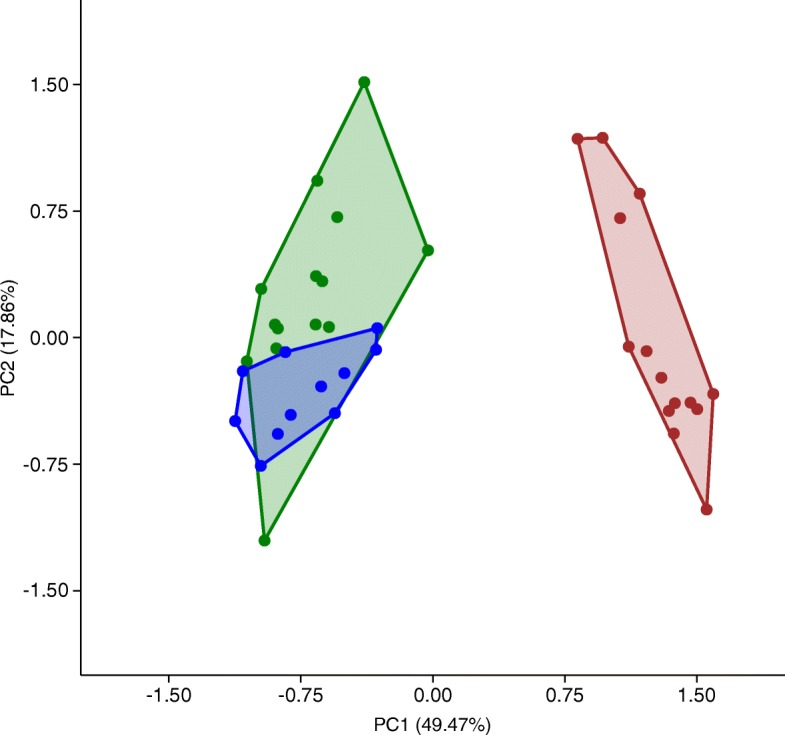
Principal Component Analysis of of the ossification sequences of 24 postcranial bones (relative ranks from 0 to 1) in 39 vertebrate species analysed in this study. Species are plotted across PC1 (49.47%) and PC2 (17.86%), and are grouped as Aves (red), Chiroptera (green), and non-volant mammals (blue)

PCA analysis revealed a clear differentiation between the prenatal ossification of the postcranium of mammals and birds, with bats and non-volant mammals occupying a single developmental space (Fig. 3). PC1 and PC2 combined explained 67.33% of the variation. Chiroptera’s development showed the highest variability in our sample, occupying the largest space across PC1 and PC2. Negative values along PC1 (49.47%) indicate delayed ossification of the caudal vertebrae, manual phalanges, pubis and ischium, whereas positive values indicate early ossification of clavicle, humerus and radius. PC2 (17.86%) is associated with early ossification of the presacral vertebrae.

### Developmental growth

In contrast to our results of sequence heterochrony in early-ossifying bones, the last bones to ossify were the ones with the shortest lengths across bat species (Additional file 3: Table S1). Such is the case for the sternum and the carpals, where for most species the adjusted ranks were frequently zero. Overall, CRL had the highest variance of all measurements, followed by UL and RL (see Table 2 for abbreviations). CaL and CavL showed the lowest variance, followed by TaL and StL. Regarding the limbs, measurements of the zeugopod showed the highest variance, whereas measurements of the autopod showed the lowest. With the exception of the cervical vertebrae, most of the sections of the spinal column had similar magnitudes of variance.

### Developmental modularity

**Fig. 4:**
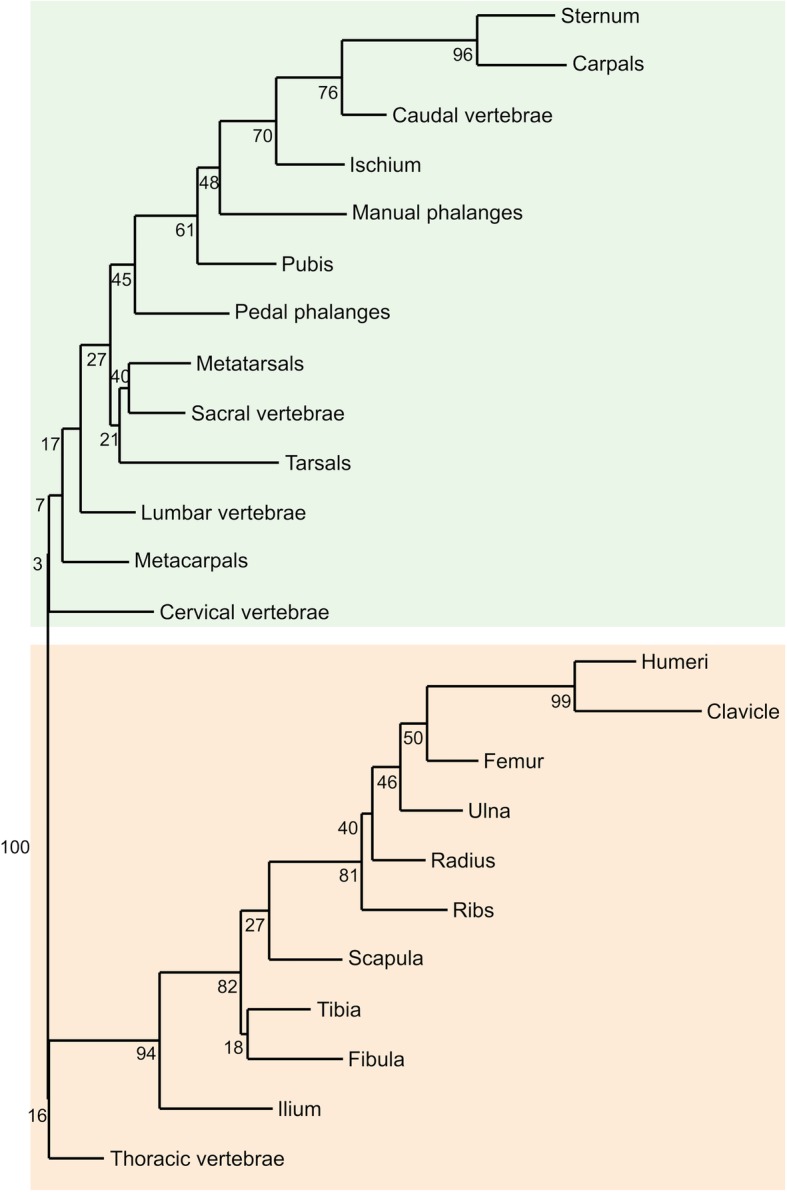
Neighbour-joining clustering analysis of ossification sequence of 24 postcranial bones (relative ranks from 0 to 1) in bats. Colours represent the two best-supported groups. Numbers represent level of support at each node after 10,000 randomisations

Permutated neighbour-joining cluster analysis revealed two well-defined clusters of bones in the postcranium, one comprising the stylopod and zeugopod of both limbs as well as both girdles and the ribcage, and the other comprising the autopod of both limbs and most of the spinal column (Fig. 4).

Of all nine modularity hypotheses tested with Kendall’s τ across all birds, bats and non-volant mammals, six were statistically significant before Bonferroni correction (Table 3). Axial and appendicular modules were significant for all taxonomic groups, whereas the forelimb and hindlimb functional modules were significant only for Chiroptera and non-volant mammals. After Bonferroni correction, however, the appendicular and axial hypotheses were both significant for Chiroptera and non-volant mammals, whereas only the appendicular was significant for birds. The abaxial hypothesis was significant for both mammal groups only.

**Table 3:** Kendall’s τ results testing nine different modularity hypotheses of metric growth

	Chiroptera	Aves	Non-volant mammals
Vertebral column (1)	0.2 (0.8065)	−0.111 (1)	0.738 (0.129)
Axial skeleton (2)	0.879 (< 0.0001)*	0.576 (0.1662)	0.806 (< 0.0001)*
Appendicular skeleton (3)	0.818 (0.0002)*	0.795 (< 0.0001)*	0.907 (< 0.0001)*
Armwing (4)	1 (1)	0.816 (1)	1 (1)
Handwing (5)	1 (1)	0.333 (1)	1 (1)
Forelimb functional (6)	**1 (0.0085)**	0.745 (0.0691)	**1 (0.0085)**
Hindlimb functional (7)	**0.714 (0.0187)**	**0.837 (0.0061)**	**0.837 (0.0061)**
Abaxial skeleton (8)	0.856 (< 0.0001)*	**0.495 (< 0.0053)**	0.775 (< 0.0001)*
Primaxial skeleton (9)	0.467 (0.2596)	0.286 (0.5593)	**0.828 (0.0354)**

*P* values shown in parentheses. Values in bold were statistically significant before Bonferroni correction, and asterisks indicate significance after correction (*P* < 0.05/29 = 0.0017)

PCA results grouping linear measures of all 24 bones based on this axial-appendicular model showed a clear distinction between the two modules, with only four variables showing an overlap between both modules (Additional file 4: Figure S3).

### Disparity and integration

**Fig. 5:**
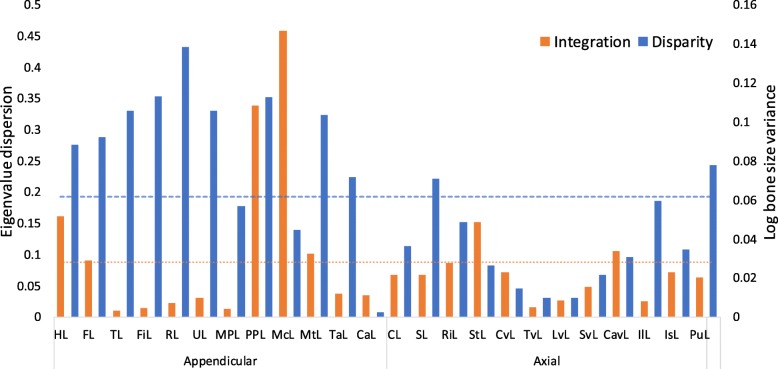
Developmental integration (eigenvalue dispersion) and disparity (bone size variance) across 24 postcranial bones in bats. Dotted and dashed lines mark average integration and disparity respectively

**Fig. 6:**
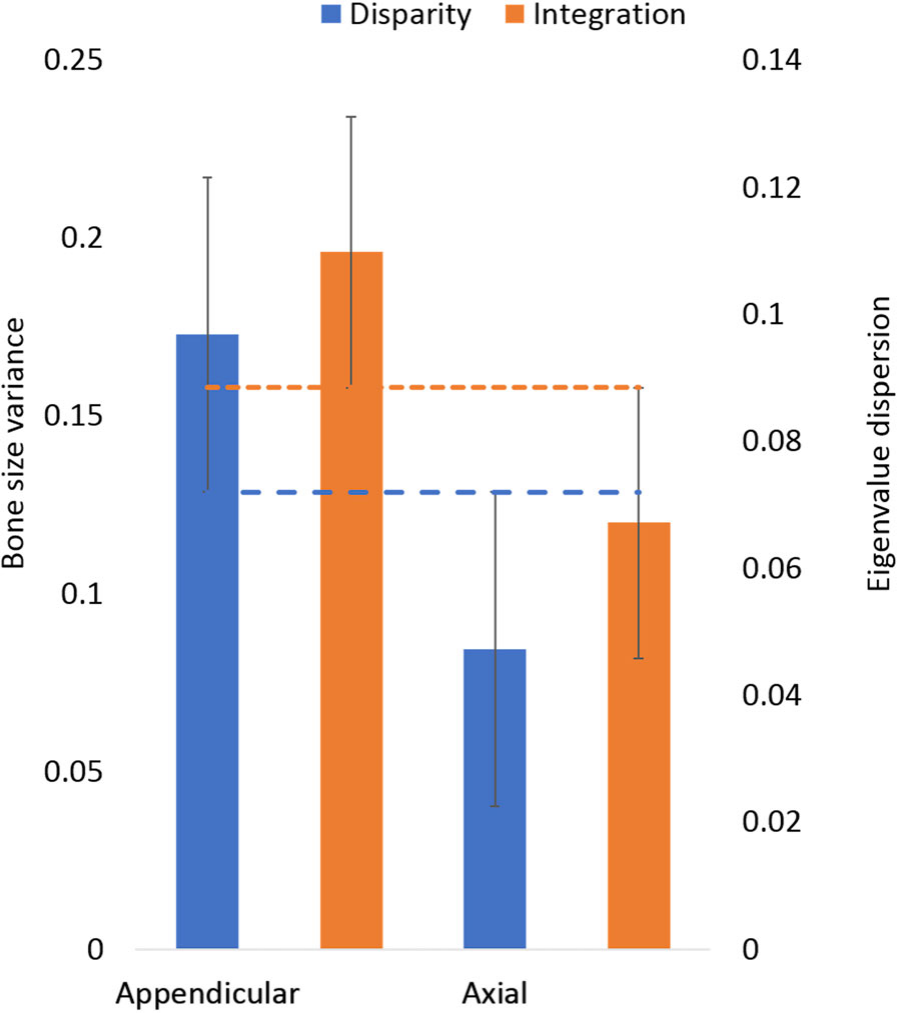
Average developmental integration (eigenvalue dispersion) and disparity (bone size variance) of elements of the appendicular and axial developmental modules in bats. Dotted and dashed lines mark intermodule mean integration and disparity respectively. Error bars indicate standard deviation

**Fig. 7:**
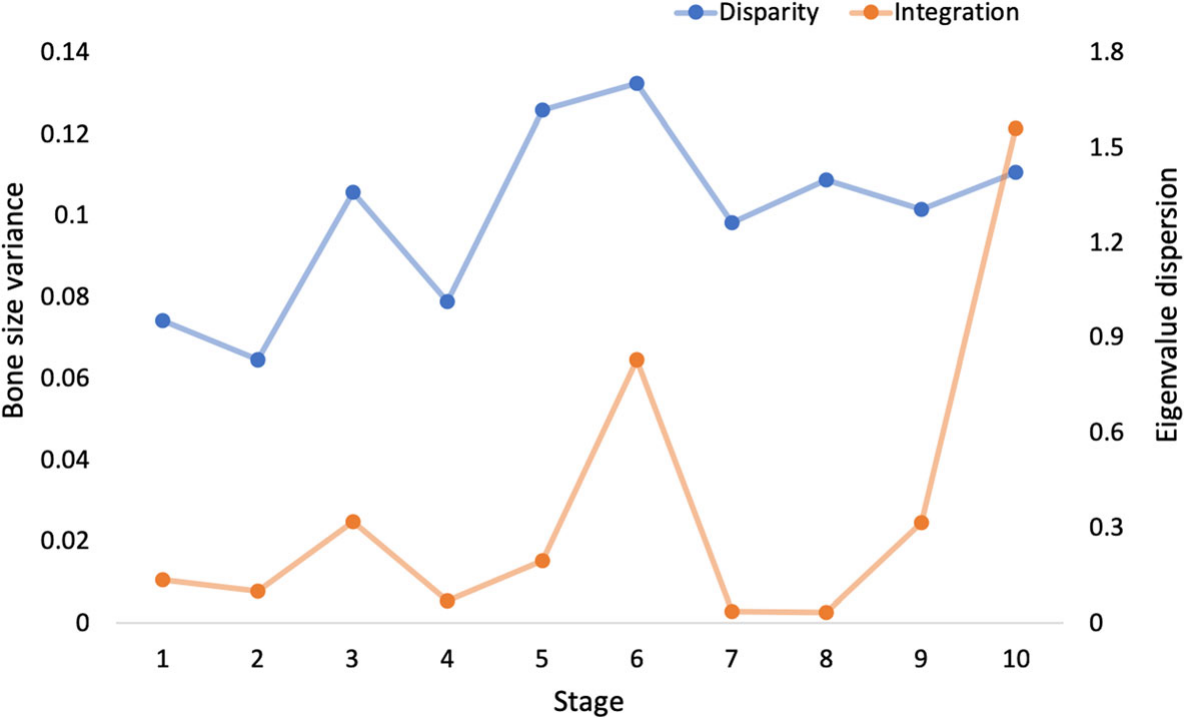
Developmental integration (eigenvalue dispersion) and disparity (bone size variance) across prenatal stages for bats examined in this study. Values at each stage are an average across all bones

Values of integration ranged from 0.011 to 0.458 across the postcranium (mean 0.088). Seven bones showed integration values higher than average, with metacarpals and pedal phalanges with the highest values (0.458 and 0.338 respectively; Fig. 5). Manual phalanges, tibia and fibula showed the lowest values (< 0.02; Fig. 5). Disparity in bats ranged from 0.002 to 0.138 (mean 0.061), with the highest values found in the radius and fibula, and the lowest values in the carpals and the thoracic vertebrae. The appendicular module showed higher values of integration and disparity than the axial module, both higher than average (0.175 for disparity and 0.11 for integration), whereas both integration and disparity in the axial module were below average (0.075 and 0.07) (Fig. 6). Disparity was significantly different between modules, whereas integration was not (ANOVA, disparity: *P* = < 0.001, *F*_(1,23)_ = 15.49; integration: 0.333, *F*_(1,23)_ = 0.978). GLM did not reveal a significant association between integration and disparity across bones (*P* = 0.832, R^2^ = 0.002; Additional file 5: Figure S4). Our results of disparity and integration across developmental time show that both disparity and integration increase across time, but neither follows a clear temporal pattern (Fig. 7).

**Fig. 8:**
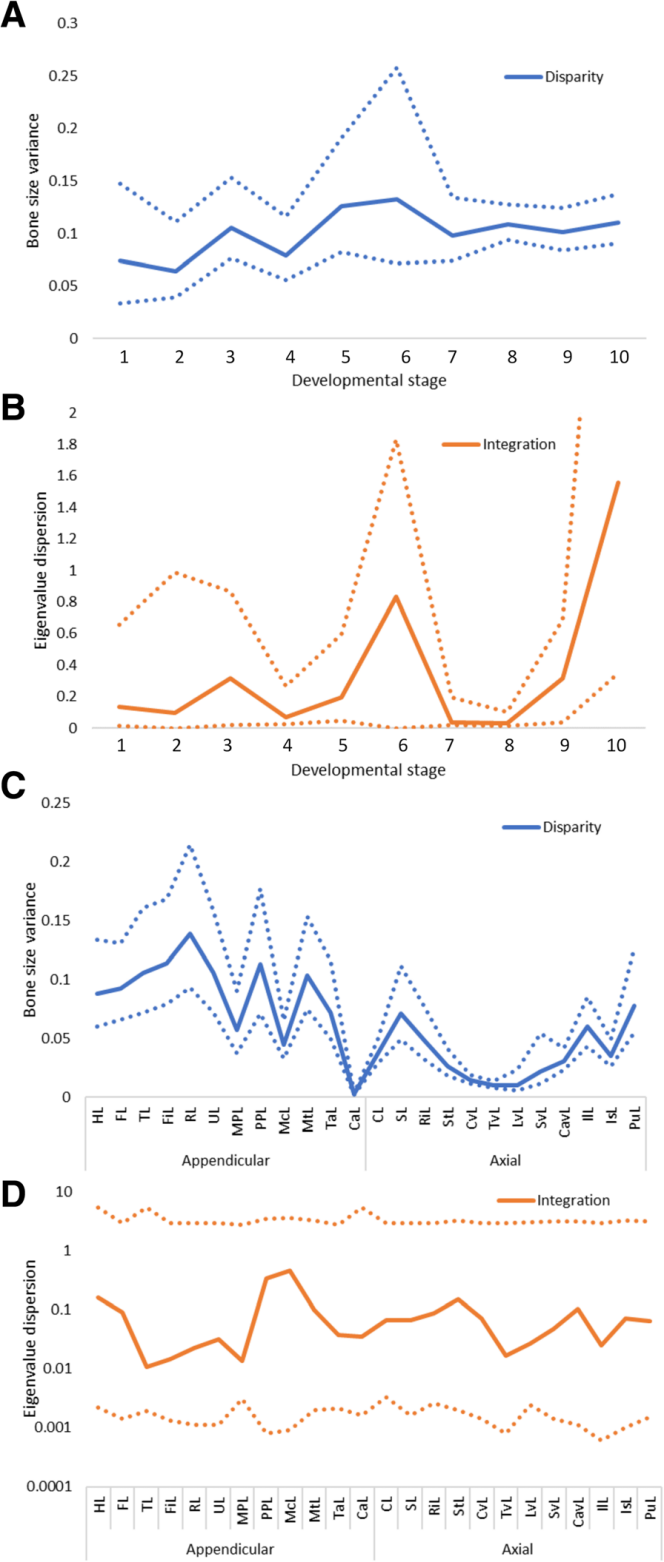
95% confidence intervals of values of disparity (**a**, **c**) and integration (**b**, **d**) across bat developmental stages (**a**, **b**) and bones (**c**, **d**) based on 10,000 bootstrap replicates

LDA showed that the staging system implemented for the bat specimens successfully separated developmental stages (93.94% of specimens correctly classified, Additional file 2: Figure S2). Stages were characterised by a CRL range and ossification events specific to each stage (Additional file 6: Table S2). Disparity across developmental stages had a tendency to increase but neither linearly nor exponentially (R^2^ = 0.31 and 0.36). Integration, on the other hand, reached its highest values in stages 6 and 10, but did not show a clear pattern of increase that fitted either a linear model or exponential curve (R^2^ = 0.26 and 0.06). Pearson’s correlations showed non-significant relationships between disparity, integration and the number of specimens per stage (disparity: *ρ* = 0.103, *P* = 0.775; integration: *ρ* = − 0.19, *P* = 0.584; Additional file 7: Figure S5). Pearson’s correlation also showed that differences in species sampling across stages had no effect in values of disparity and integration (disparity: *ρ* = 0.006, *P* = 0.986; integration: *ρ* = − 0.228, *P* = 0.525). GLM showed that integration and disparity are not correlated across developmental time (*P* = 0.199, R^2^ = 0.196; Additional file 8: Figure S6). Bootstrapping revealed that all disparity and integration values (both across bones and developmental stages) fall within the 95% confidence intervals, indicating statistical significance (Fig. 8).

## Discussion

### Sequence heterochrony and developmental growth

The ossification of the clavicle is consistently the first event across birds and mammals, a pattern well-known for vertebrates [66]. Compared with birds, mammals (both bats and non-volant mammals) show a delayed ossification of the stylopod and zeugopod of both limbs, indicating that stylopod and zeugopod development in bats resembles the general pattern found in Mammalia [36]. Patterns in our results support heterochrony between the forelimb and hindlimb in bats and birds, compared to non-volant mammals (Fig. 2). Similarities between bats and birds in ossification sequences of pedal phalanges could indicate a developmental response to locomotory needs of newborns [36, 77]. Despite behavioural differences in the neonates, and because newborn bats and birds are incapable of self-powered flight, locomotion and roosting depend mostly on hindlimb functional performance [36, 77]. Based on recent palaeontological findings suggesting newborn pterosaurs were incapable of flight [92], we suggest that hindlimb heterochrony could be a trait shared across flying vertebrates [36, 77]. Our results support the hypothesis that accelerated development of the foot in bats correlates with the roosting ecology of newborn pups, which cannot fly and need to remain attached to either the mother or the roosting site at all times, until they achieve self-powered flight [36]. In contrast, delayed development of the carpals could be related to the functional importance of the wrist for the folding of the wing during the upstroke, a key kinematic process during flight [93]. Bats also show accelerated ossification of the jaw for suckling and attachment to the mothers’ nipples during flight [36].

Marked heterochrony in the ossification of the sternum of bats and birds could indicate an ontogenetic basis for flight altriciality in flying vertebrates. The presence of a sternal ridge or keel in the sternum is a crucial adaptation for flight in vertebrates, as it provides additional surface for the attachment of muscles involved in wingbeat movements [94]. Delayed ossification and longer developmental times have recently been proposed to be associated with evolvability and adaptability, although thus far only within the context of domestication [58, 98]. Our findings of delayed sternum ossification along with its importance for locomotion across flying vertebrates [94, 95], may also be fitting with that hypothesis [58]. Finally, unsynchronised ossification of the pelvic bones reflects the different chondrogenous centres that lead to the formation of the hip as a single structure [96], a pattern previously seen in other mammalian groups but not in birds [34, 36, 77].

PCA of ossification sequences reveals a clear separation between mammals and birds along the first principal component (PC1), showing that phylogenetic relationships shaped our results (Fig. 3). Based on the apparent mammal (negative loadings) to bird (positive loadings) polarity across PC1, it can be argued that bones with positive loadings exemplify bird development, whereas bones with negative values exemplify mammal development. All sections of the vertebral column had negative loadings, whereas the stylopod and zeugopod of both limbs had positive loadings, indicating that axial skeleton development differentiates mammal development, whereas appendicular skeleton development differentiates bird development (Additional file 9: Table S3).

Similarities between birds and bats in postcranial development suggest an ontogenetic basis to the convergent evolution of vertebrate flight. Our results show some similarities with recent palaeontological findings that informed the prenatal development of pterosaurs [92]. It is hypothetised that newborn pterosaurs were incapable of self-powered flight due to an altricial wing, a trait shared with bats and birds [36, 77, 92]. Moreover, based on our results, we can suggest a shared ontogenetic basis to flight altriciality, as a general delay in the ossification of the wing and sternum compared with the hindlimb is found across all flying vertebrate groups [36, 77, 92]. Relative timing of morphogenesis and incomplete prenatal development could act as an evolutionary promoter of adaptability and evolvability in the forelimb of flying vertebrates, facilitating flight adaptations to evolve [58]. Previously, prenatal developmental timing has been associated with evolvability in cranial shape of carnivorans, also showing an ontogenetic source for variability associated with domestication in canids [58, 97, 98].

Our results also showed that the development of the vertebral column of bats deviates from both the non-volant mammals and birds. In comparison to other mammals, unique morphological features found in the vertebral column of bats have been linked to the roosting and feeding ecology of the species [99]. Our findings of sequence heterochrony in the vertebral column indicate that the morphological patterns found in this region in previous studies could have an ontogenetic basis, originating during the prenatal development of the skeleton [47, 99].

Evolutionary changes in bone size have been correlated with heterochronic development of those structures [100]. Our result that bones that grow to be relatively small in the adult have a late ossification onset does not reflect this phenomenon (Fig. 5). Instead, our results could reflect that at the stage sampled much of the growth for the elements examined here was yet to be completed (i.e. postnatally), leading to a narrower depiction of its total growth path [101]. Future studies could combine pre- and postnatal developmental data to trace the complete development of the hip.

### Developmental modularity

Our analysis did not reject the hypothesis that postcranial developmental modularity is present in bats, birds and non-volant mammals (Figs. 3, 4, Table 3). The presence of an axial-appendicular partition in both mammal groups, not found in birds, could indicate that flight did not repattern ontogenetic modularity in flying vertebrates, as bats and non-volant mammals shared the same pattern for postcranial modularity. Furthermore, our results supported the presence of an appendicular skeleton module, previously unknown in bats [102], and an axial module previously found in placental prenatal development [102]. We suggest a developmental module hypothesis, where the shared LPM-derived origin of all bones of the appendicular skeleton develop as a unit [103].

Modularity in the appendicular skeleton of mammals has been reported to respond to functional pressures and selection on short times scales, as in domestication [104, 105]. Presence of developmental modularity in mammals has revealed a clear ontogenetic division between marsupials and placentals, each group presenting different patterns of postcranial modularity [102]. In particular, placental mammals showed strong evidence of an appendicular module and a module including both girdles [102]. However, previous studies suggested that bats are an exception to this trend, showing low covariation between hind- and forelimb both during their development and adulthood [23, 102, 106, 107], in contrast to our results. Each of these studies focused on modularity in mammals as a whole, including only one bat species in their sample. The contrasting results could be due to differences in sample composition, with low bat species representation obscuring patterns only discernible when analysing multiple species representing different lineages. With 14 bat species, our study represents the most comprehensive quantitative study of prenatal developmental biology in chiropterans to date. Also, our results of developmental modularity were informed by two different datasets that combined sequence heterochrony and metric growth data, contrary to previous studies that focused only on one.

Our two sets of results could indicate that the functional differences between the forelimb and hindlimb in adult bats are not reflected during prenatal development [15, 108]. Moreover, integrated development of homologous structures could facilitate morphological diversity, enabling novel functional ecologies to evolve [46, 59, 62, 109]. The latter may indicate that the correlated development of both limbs in bats could facilitate morphological disparity in adult forms. In bats, neonate hindlimb size is similar to its adult size whereas neonate forelimb size is about one third of adult size [36]. Since the forelimb is not developed sufficiently for flight at birth and requires extended postnatal time to be large enough to be fully functional, it was suggested that prenatal bats invest in earlier development of the hindlimb [36]. Given the present results, we hypothesise that modular development in the appendicular skeleton could represent a trade-off between the accelerated development of the foot (and functionality in newborns), and the delayed development of the wing (and its functionality in later development; [93]). Delayed prenatal development has been linked to higher adaptability and evolvability to environmental pressures in domesticated carnivorans [58].

### Disparity and integration

High levels of disparity in the zeugopod of both limbs document the developmental basis of its functional variability (Fig. 4). Compared to the ancestral mammalian condition, zeugopod reduction is a convergent trait common across multiple clades [110]. This reduction arguably enabled locomotory specialisations that led to the adaptive radiation of mammals [110]. Diminished growth rate of the zeugopod – in particular the ulna and fibula – is a developmental process that has been found in several different mammal groups, arguing for a shared ontogenetic mechanism for zeugopod reduction [110]. Our results indicate that zeugopod development is highly variable across bat species, possibly reflecting functional differences associated with ecological traits of the species (e.g. flying behaviours, locomotory specialisations, and body sizes) [32, 111, 112]. Forearm length is widely used to inform field identification of bat species, providing a good indicator of the interspecific morphological delimitation in many taxa [113]. Additionally, relative forearm length is also a good estimate of several biomechanical properties of the wing that reflect ecological differences across species [114]. We hypothesise that high prenatal disparity of the zeugopod could be indicative of ecomorphological diversity in adult bats.

Our PCA of species could reflect functional differences by showing that *C. sphinx* (the only plant-eater and non-echolocating species in our sample for this analysis) was the most dissimilar (Additional file 10: Figure S7 and Additional file 11: Table S4). Plant-visiting bats exhibit foraging behaviours uncommon in animalivorous species (e.g. prolonged hovering flight) that could represent novel functional needs for the postcranium. However, the PCA also shows that the subordinal spaces did not overlap, raising the question of whether our results evidence a phylogenetic signal, rather than functional. Descriptions of the broad foraging guilds for these bat families suggest that all animalivorous species in our sample are aerial hawkers [115], indicating a phylogenetic signal in our results. Nevertheless, the ecology of some of the species is either poorly known (e.g. *Aselliscus*) or highly adaptable (e.g. *Kerivoula* and *Myotis* species are known to switch between hawking and gleaning) [116, 117]. Expanding the sample to include frugivorous yangochiropteran species (i.e. phyllostomids) could yield further insights.

Phylogenetic studies have reconstructed clade-specific evolutionary trajectories of joint and muscle reduction in the wings of bats, closely associated with flying behaviours in response to feeding strategies [118]. Such correspondence between ecological diversity and developmental differences has been previously reported in other mammals [47]. Finally, low levels of integration in the zeugopod and a generalised increase in disparity indicate that integration could promote disparity during prenatal development [59].

Differences in disparity between modules also illustrate the ontogenetic basis of the phenotypic diversity and functional variability of the limbs in bats. Prenatal development has been suggested to respond to the functional needs of the neonates, rather than the ecological niche of the species [36]. As such, lower than average disparity in the axial module suggests that this section of the skeleton does not experience high evolutionary pressure to diversify prenatally [15, 36, 119, 120].

Our results provide strong evidence for increase in disparity over ontogeny (Fig. 7), a pattern rarely reported in mammals [59]. Moreover, ours represents the first report of this pattern both in bats and in prenatal development. Previous studies have shown that integration canalises phenotypic variation in deep-time [59, 121–124], and that changes in size can work as evolutionary buffers for adaptive radiation [125]. Differences between morphological canalisation over deep time and our results across developmental time support the hypothesis that integration facilitates disparity from an ontogenetic perspective [59, 121]. This congruence in temporal patterns differs from the mismatch in patterns of integration and disparity found across bones and modules, demonstrating that temporality rather than functionality shapes the interaction between developmental disparity and integration [49, 59].

## Conclusions

Combining data of sequence heterochrony and developmental growth from 14 bat species, this study represents the most comprehensive quantitative analysis of chiropteran prenatal ossification to date. Sequence heterochrony between the autopod of fore- and hindlimbs could reflect functional needs of the newborn, rather than ecological aspects of the adult. Sequence heterochrony also showed that bats have similarities with birds in the ossification of structures involved in flight (i.e. handwing and sternum), suggesting that flight altriciality and early ossification of pedal phalanges and sternum are common across flying vertebrates. Developmental modularity was detected both in ossification sequence and metric growth of the postcranium, suggesting an axial-appendicular partition of the postcranium that deviates from the general pattern reported for mammals. This partition possibly corresponds to genetic and ontogenetic aspects of the development of the postcranium (e.g. the LPM-derived nature of all appendicular bones), rather than ecomorphological characteristics of bats. The marked difference in values of disparity between modules reflects the phenotypic diversity of the appendicular skeleton in response to interspecific functional differences of the wing. Our results reject the hypothesis that morphological variation in the fore- and hindlimbs of bats is dissociated. Integration and disparity increased across prenatal stages, supporting the hypothesis that integration facilitates disparity. Moreover, we found that this interaction is only evident from a temporal perspective (i.e. across developmental time) rather than from a morphofunctional one (i.e. between functional modules of the skeleton). Finally, our results show not only an increase in disparity across developmental time, but also a delayed ossification in highly adaptable and evolvable regions, both patterns rarely reported in wild mammals.

## Additional files

Additional file 1: **Figure S1.** Flowchart summarising the methodology used in our study. (TIFF 363 kb)

Additional file 2: **Figure S2.** LDA of staging system implemented for bats in this study. (TIFF 266 kb)

Additional file 3: **Table S1.** Average values (mm) of 25 postcranial linear measurements of 66 specimens of 11 bat species and all species pooled. Standard deviation in parenthesis. (CSV 3 kb)

Additional file 4: **Figure S3.** PCA of linear measurements of 24 postcranial bones in bat foetuses. Bones are grouped reflecting the axial and appendicular modules found in our Kendall’s τ modularity analysis. (TIFF 533 kb)

Additional file 5: **Figure S4.** Generalized Linear Model (GLM) of integration (Eigenvalue dispersion) and disparity (bone size variance) values across all 24 bones measured and all bat species pooled. Each point represents a single skeletal element. (PNG 18 kb)

Additional file 6: **Table S2** Description of developmental stages 1–10 based on CRL ranges and ossification events that characterise each stage. “X” represents the ossification onset of a given bone. (CSV 1 kb)

Additional file 7: **Figure S5.** Scatterplot of integration (Eigenvalue dispersion) and disparity (bone size variance) values in bats against number of specimens in each stage. (PNG 19 kb)

Additional file 8: **Figure S6.** GLM of integration (Eigenvalue dispersion) and disparity (bone size variance) values across developmental time in bats examined. Each point represents a developmental stage. (PNG 15 kb)

Additional file 9: **Table S3.** PCA loadings of relative ossification rank for 24 postcranial bones. (CSV 1 kb)

Additional file 10: **Figure S7.** PCA of bat species based on metric growth data. Ellipses show the developmental space of both suborders. (PNG 594 kb)

Additional file 11: **Table S4.** Bone size variance (disparity) and eigenvalue dispersion (integration) values of 24 postcranial bones of nine bat species included in this study. Left and right boxes include bones of the appendicular and axial module respectively. Int = Integration, Disp = disparity. (CSV 2 kb)

Additional file 12: Ossification sequence data used in this study. (XLS 40 kb)

Additional file 13: Metric growth data used in this study. (XLSX 17 kb)

## Abbreviations

CaL: Carpal Length
CavL: Caudal Vertebral Width
CL: Clavicular Length
CRL: Crown to Rump Length
CvL: Cervical Vertebral Width
FiL: Fibular Length
FL: Femoral Length
GLM: Generalised Linear Model
HL: Humeral Length
IlL: Ilium Length
IsL: Ischium Length
LDA: Linear Discriminant Function Analysis
LPM: Lateral Plate Mesoderm
LvL: Lumbar Vertebral Width
McL: Metacarpal Length
MPL: Manual Phalanges Length
MtL: Metatarsal Length
PCA: Principal Component Analysis
PPL: Pedal Phalanges Length
PuL: Pubis bone Length
RL: Radial Length
RL: Rib Length
SL: Scapular Length
StL: Sternum Length
SvL: Sacral Vertebral Width
TaL: Tarsal Length
TL: Tibial Length
TvL: Thoracic Vertebral Width
UL: Ulnar Length
