# Supplementary figures and images for: Postcranial heterochrony, modularity, integration and disparity in the prenatal ossification in bats (Chiroptera)
Source: BMC Evol Biol. 2019 Mar 12;19:75. doi: 10.1186/s12862-019-1396-1 (PMC6417144; doi:10.1186/s12862-019-1396-1)

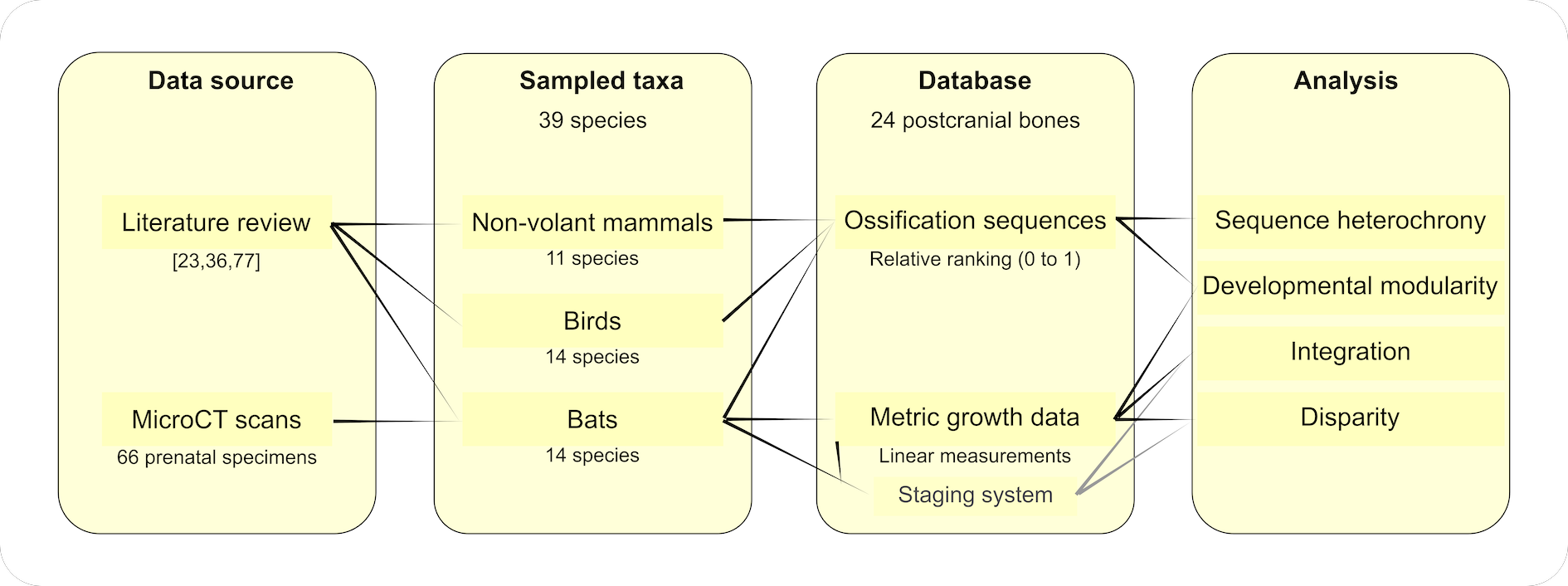

Supplement: Supplementary file 1 — Figure S1. Flowchart summarising the methodology used in our study. (TIFF 363 kb) [file 12862_2019_1396_MOESM1_ESM.tiff]

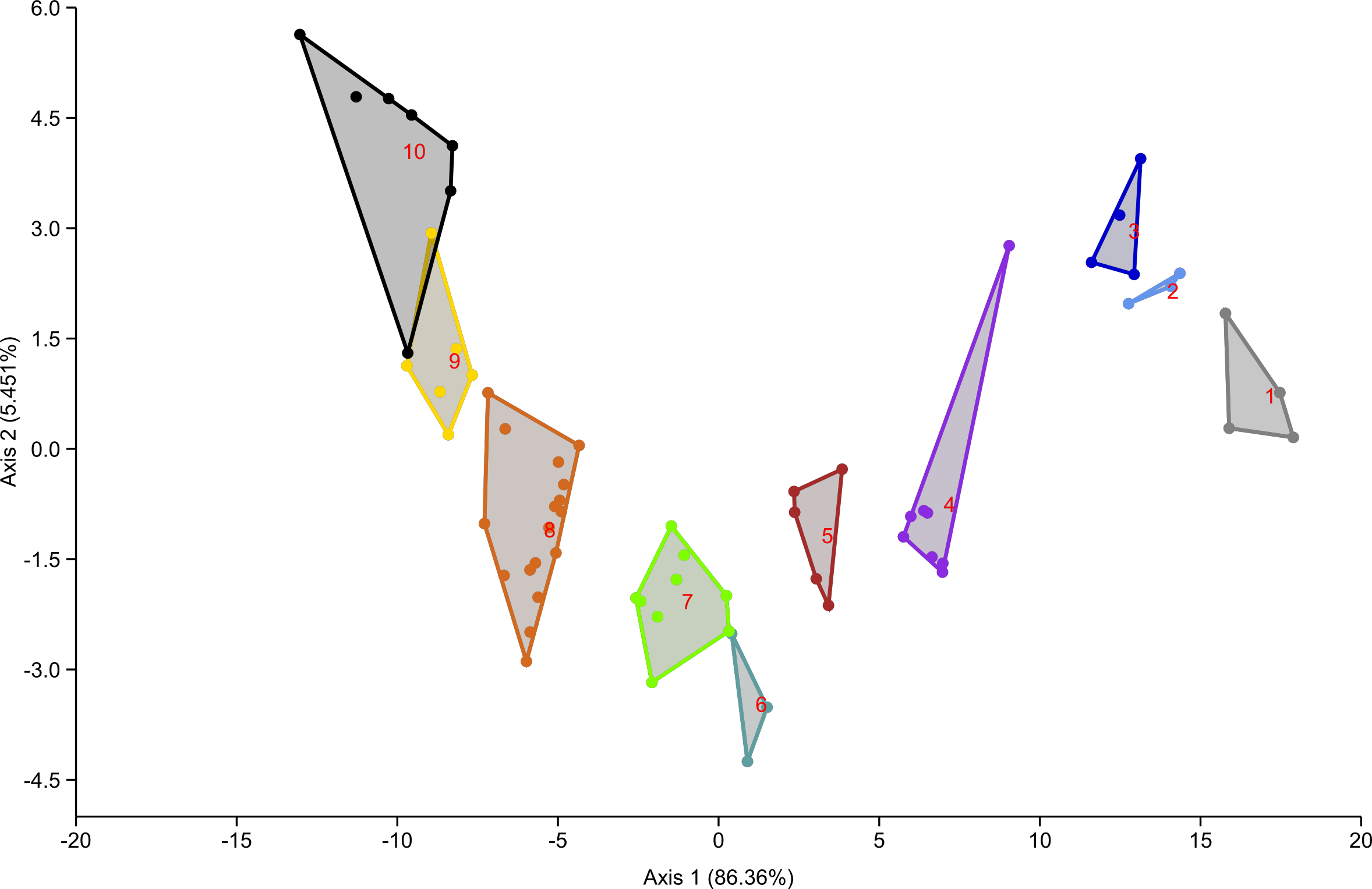

Supplement: Supplementary file 2 — Figure S2. LDA of staging system implemented for bats in this study. (TIFF 266 kb) [file 12862_2019_1396_MOESM2_ESM.tiff]

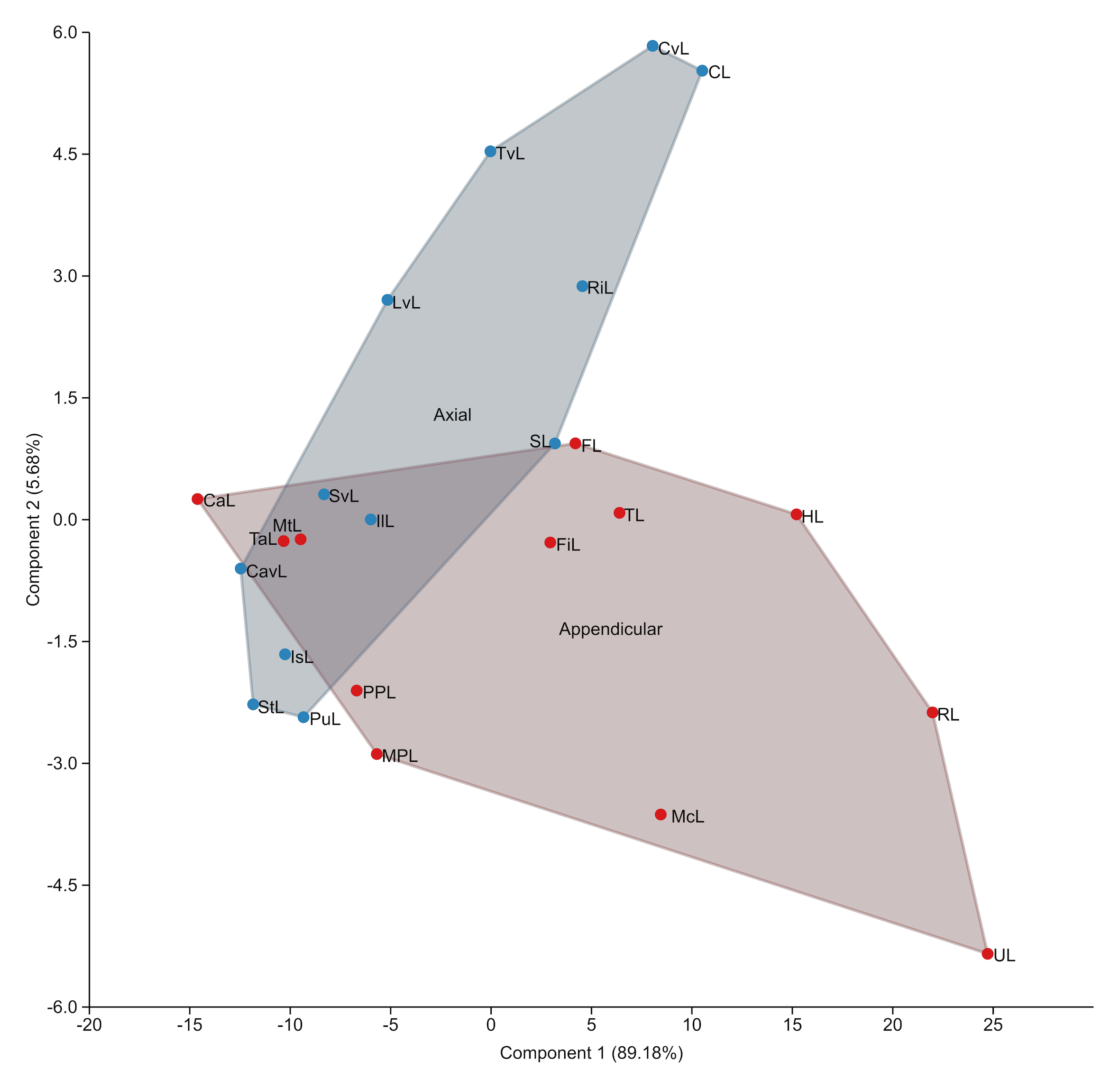

Supplement: Supplementary file 4 — Figure S3. PCA of linear measurements of 24 postcranial bones in bat foetuses. Bones are grouped reflecting the axial and appendicular modules found in our Kendall’s τ modularity analysis. (TIFF 533 kb) [file 12862_2019_1396_MOESM4_ESM.tiff]

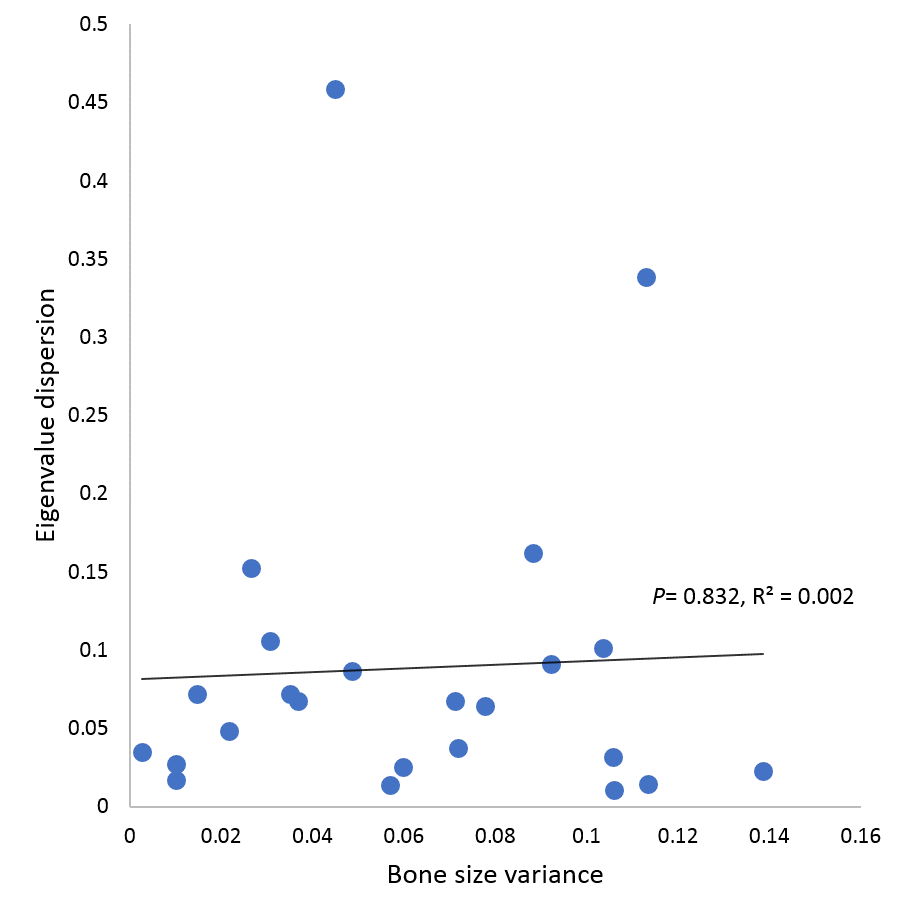

Supplement: Supplementary file 5 — Figure S4. Generalized Linear Model (GLM) of integration (Eigenvalue dispersion) and disparity (bone size variance) values across all 24 bones measured and all bat species pooled. Each point represents a single skeletal element. (PNG 18 kb) [file 12862_2019_1396_MOESM5_ESM.png]

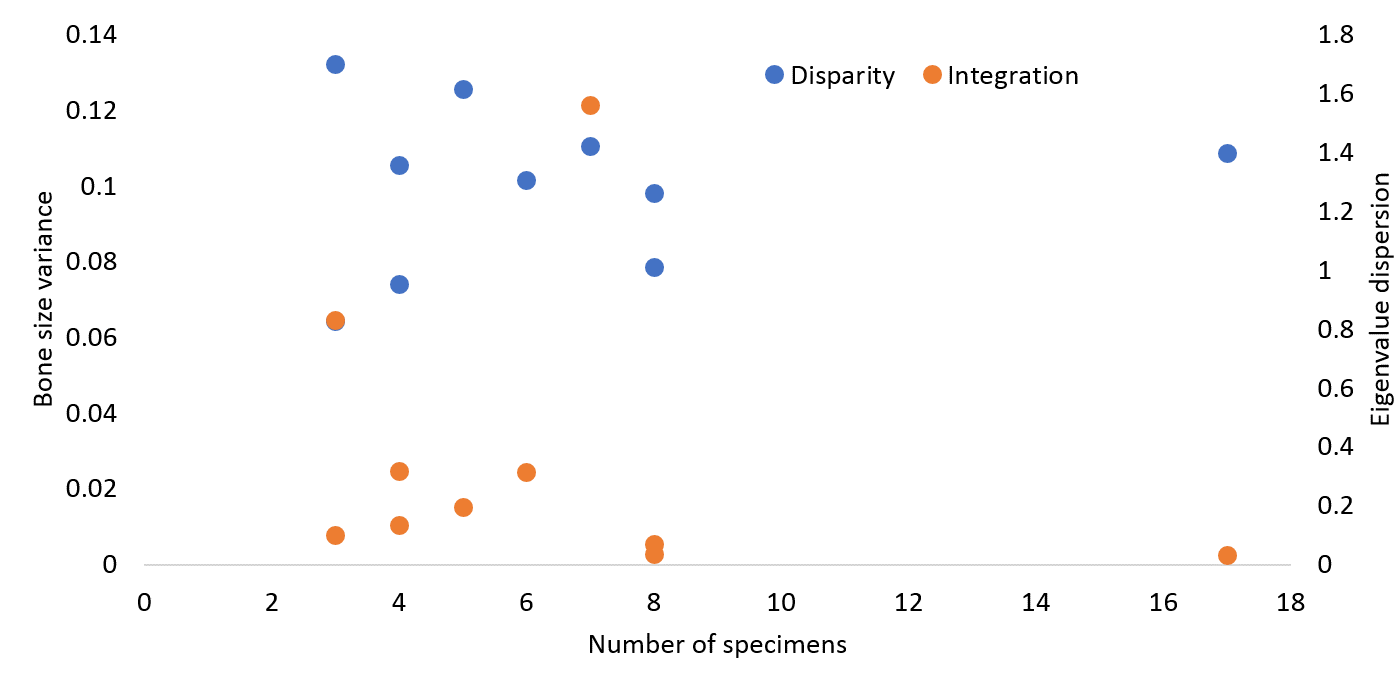

Supplement: Supplementary file 7 — Figure S5. Scatterplot of integration (Eigenvalue dispersion) and disparity (bone size variance) values in bats against number of specimens in each stage. (PNG 19 kb) [file 12862_2019_1396_MOESM7_ESM.png]

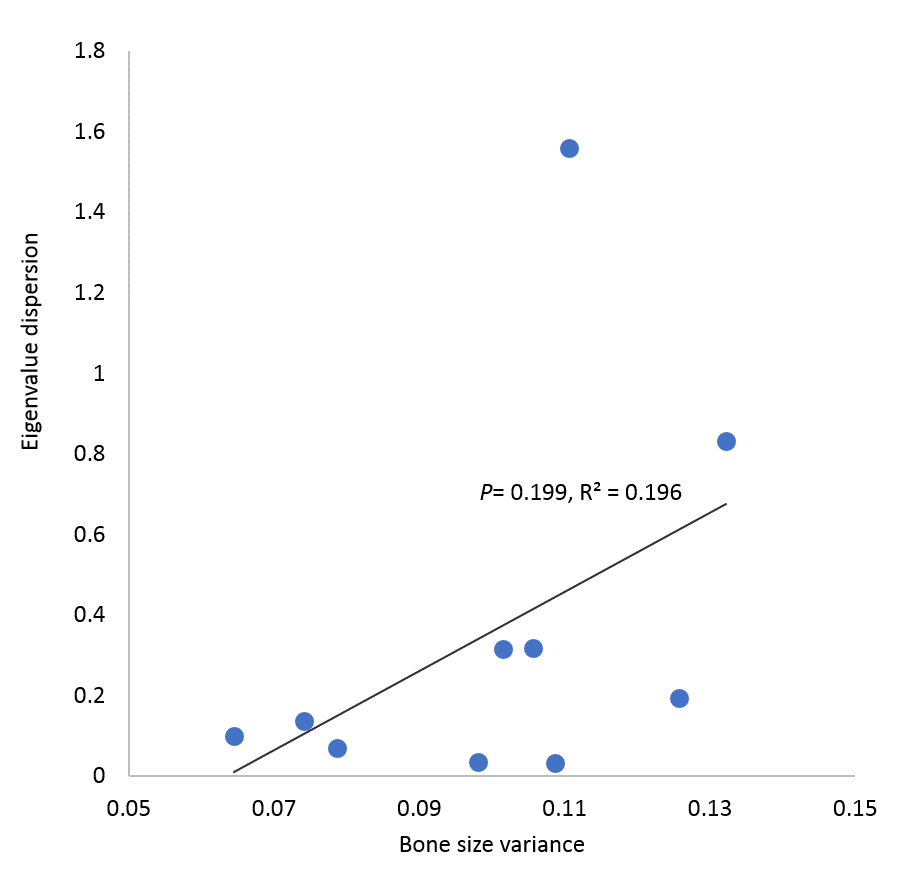

Supplement: Supplementary file 8 — Figure S6. GLM of integration (Eigenvalue dispersion) and disparity (bone size variance) values across developmental time in bats examined. Each point represents a developmental stage. (PNG 15 kb) [file 12862_2019_1396_MOESM8_ESM.png]

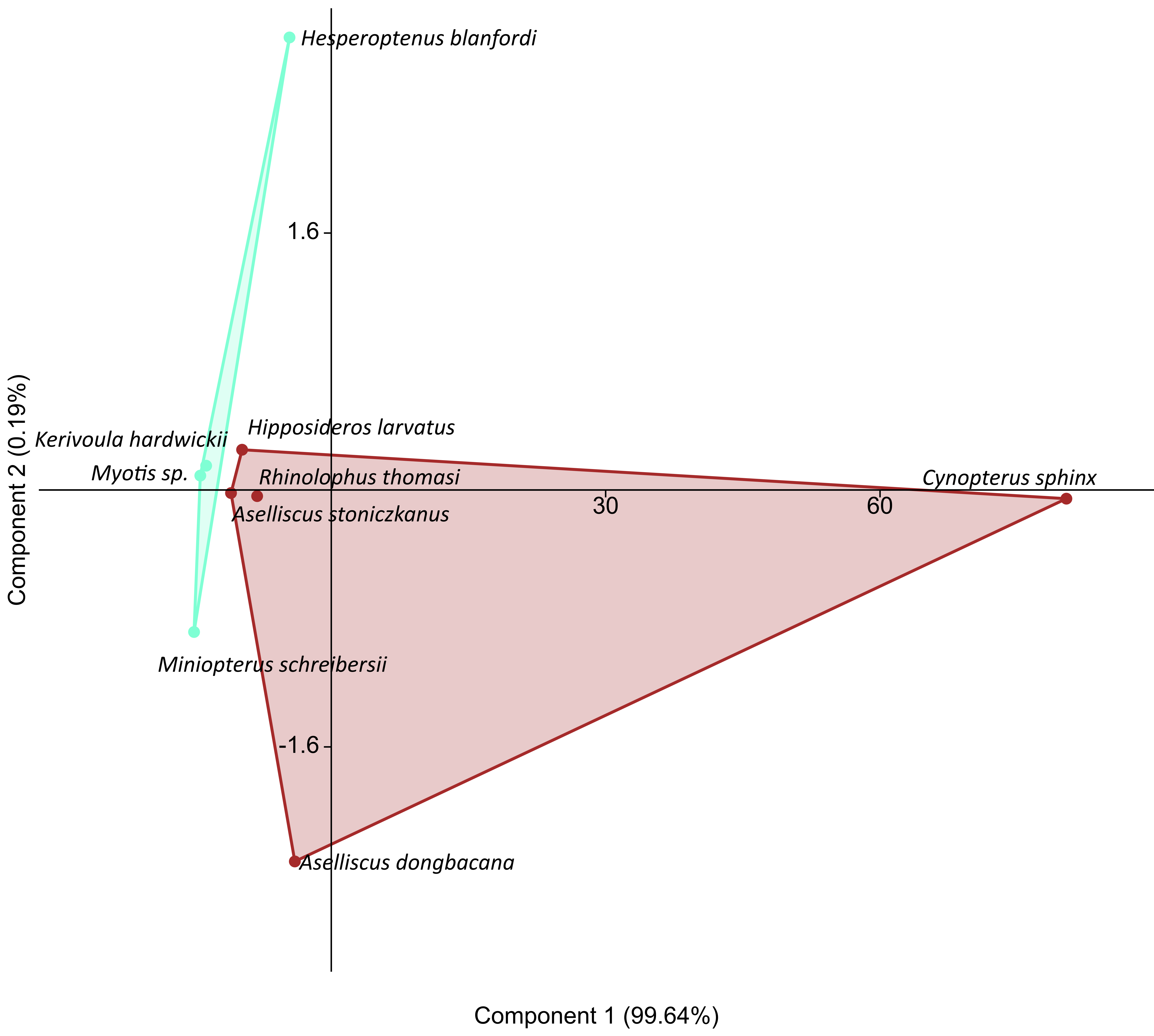

Supplement: Supplementary file 10 — Figure S7. PCA of bat species based on metric growth data. Ellipses show the developmental space of both suborders. (PNG 594 kb) [file 12862_2019_1396_MOESM10_ESM.png]
